# LHPP Inhibits the Proliferation and Metastasis of Renal Cell Carcinoma

**DOI:** 10.1155/2020/7020924

**Published:** 2020-12-22

**Authors:** Xiaoting Zhang, Huaning Kang, Jing Xiao, Benyan Shi, Xiaofeng Li, Guihong Chen

**Affiliations:** ^1^Shenzhen Bao'an District Songgang People's Hospital, Shenzhen 518100, China; ^2^School of Pharmaceutical Sciences, Guangzhou Medical University, Guangzhou 511436, China; ^3^Department of Laboratory Medicine, Peking University Shenzhen Hospital, Shenzhen, Guangdong Province, China

## Abstract

Renal cell carcinoma (RCC) is one of the ten most common cancers in the globe. Despite the diagnosis and treatment of renal cell carcinoma that have made great improvements, the morbidity and mortality rates of renal cell carcinoma remain unchanged remarkably. LHPP is a kind of histidine phosphatases, acting as a tumor suppressor in the progression of various cancers. In this study, we found that LHPP was significantly downregulated in RCC tissues and cell lines. Decreased expression of LHPP was closely correlated with tumor size and postoperative metastasis of RCC patients. In addition, overexpression of LHPP inhibited the proliferation and metastasis of RCC. However, suppression of LHPP promoted the proliferation and metastasis of RCC. In conclusion, our results presented the important role of LHPP in the development and progression of RCC.

## 1. Introduction

Renal cell carcinoma (RCC) is one of the leading causes of death among urologic neoplasm patients. Globally, it was estimated that there are 403,262 RCC new cases and 175,098 cancer-related deaths in 2018 [1]. Approximately 30% of RCC patients progress into an advanced stage at first diagnosis [2]. The 5-year survival rate of early stage RCC patients is higher than 90%, while it decreased to 10% in advanced stage RCC patients for the resistance to chemotherapy and radiation therapy [3, 4]. Therefore, it is urgent to clarify the molecular mechanisms involved in the initiation and progression of RCC and investigate effective therapeutic target for RCC.

Phosphohistidine phosphate inorganic pyrophosphatase (LHPP), a kind of histidine phosphatases, is originally discovered in swine brain tissue [5, 6]. A previous study suggested that LHPP acted as a tumor suppressor in various cancers, such as hepatocellular carcinoma, cervical cancer, bladder cancer, pancreatic cancer, and melanoma. In hepatocellular carcinoma (HCC), decreased expression of LHPP is positively correlated with larger tumor size and reduced overall survival [7, 8]. Moreover, LHPP inhibits the proliferation, migration, and invasion of hepatocellular carcinoma via decreasing the expression of MMP7, MMP9, CCNB1, and PKM2. In melanoma, overexpression of LHPP inhibits cell proliferation in vitro and in vivo [9]. In cervical cancer, high expression is closely correlated with smaller tumor size, better overall survival, and decreased lymph node metastasis. Forced expression of LHPP inhibits cell proliferation and metastasis and promotes cell apoptosis via inhibiting PI3K/AKT signal pathway activation [10]. In thyroid cancer, increased expression of LHPP represses cell proliferation and metastasis via regulating AKT/AMPK/mTOR signaling pathways [11]. However, the biological function of LHPP in RCC remains unknown.

In this study, we discovered that LHPP was significantly downregulated in RCC tissues and cell lines. In addition, decreased expression of LHPP was positively correlated with tumor size and postoperative metastasis of RCC patients. Further experiments demonstrated that augmented expression of LHPP significantly inhibited the proliferation, migration, and invasion of RCC cells. However, suppression of LHPP causes opposite effects. Hence, our results suggested that LHPP could act as a potential therapeutic target for RCC.

## 2. Material and Methods

### 2.1. RCC Tissues

In total, 72 pairs of RCC tissues and corresponding adjacent normal bladder tissues were collected from Peking University Shenzhen Hospital from 2013 to 2018. All human tissue samples were obtained with informed consent. This study was approved by the ethics committee institution of Songgang People's Hospital.

### 2.2. Cell Lines

All cells used in this study were purchased from the American Type Culture Collection (Manassas, VA). All cells were grown in Dulbecco's modified Eagle's medium (DMEM) mixed with 1% penicillin-streptomycin and 10% fetal bovine serum. All cells were grown in a 5% CO_2_ incubator at 37°C.

#### 2.2.1. Quantitative Real-Time PCR

Total RNA from RCC cell lines and RCC tissues was extracted by utilizing TRIzol reagent (Thermo Scientific, USA). Total RNAs were reversed by using a reverse transcription kit (TAKARA, Japan). Quantification of mRNA was measured by using the Real-time PCR Master Mix (TAKARA, Japan). This reaction was carried out by using a Roche LightCycler® 480II PCR instrument (Basel, Switzerland). GAPDH was used as an internal standard control. The relative RNA expression levels were calculated by the 2^–*ΔΔ*CT^ method.

#### 2.2.2. Cell Transfection

Short hairpin RNA (shRNA) targeting LHPP was obtained from GenePharma (Suzhou, China). pcDNA3.1-LHPP was ordered from GenePharma (Suzhou, China). The qRT-PCR assay was used to detect the effects of silencing and overexpression of LHPP. Both oligonucleotides and plasmids were transfected into the RCC cell lines using Lipofectamine 3000 (Invitrogen, USA).

### 2.3. Cell Proliferation Assay

The CCK-8 assay and colony formation assay were used to detect the proliferation of RCC cells. For the colony formation assay, 1000 transfected RCC cells were seeded in 6-well plates per well and incubated for 14 days. Finally, the cells were stained with 0.1% crystal violet and photographed. The stained cells were washed by using 33% glacial acetic acid. The absorbance of scrubbing solution was measured at 550 nm using a microplate reader. For the CCK-8 assay, the transfected RCC cells were grown in a 96-well plate until cell attachment. The absorbance in each well was calculated by using a microplate reader (Bio-Rad, USA).

### 2.4. Cell Migration Assay

The migration ability of RCC cells was detected by wound healing and transwell assay.

For wound healing assay, the transfected RCC cells were seeded in a 6-well plate and grown to 100% confluence. A clear wound in the cell layer was created by a 200 *μ*l pipette tip. The migrated RCC cells were observed and photographed at 0 h and 24 h after creating a wound. For the transwell migration assay, the transfected RCC cells were seeded in the upper chamber, while the lower chamber was filled with 600 *μ*l of DMEM with 10% FBS. After incubation for 48 h, the migrated cells were stained with 0.1% crystal violet solution for 15 min and photographed.

### 2.5. Cell Invasion Assay

The upper transwell chamber was covered with matrigel mix (BD Biosciences, USA) for transwell invasion assay. The transfected RCC cells were harvested and seeded in the upper chamber, while the lower chamber was filled with 600 *μ*l of DMEM with 10% FBS. After incubation for 48 h, the invasive cells were stained with 0.1% crystal violet solution for 15 min and photographed.

### 2.6. In Vivo Assay

The tumor xenotransplantation assay was performed in accordance with the requirement of the ethics committee institution of Songgang People's Hospital. Ten 4-week-old BALB/c nude mice were randomly separated into the NC group and the pcDNA3.1-LHPP group. Approximately 6 × 10^6^ 786-O cells were injected into the back of the mice. The volume of all transplanted tumors was calculated by digital calipers every week. Finally, all the mice were sacrificed and the xenograft tumors were weighted after injection.

### 2.7. Statistical Analyses

All data from three repeated experiments were presented as mean ± standard deviation (SD). Data analyses were performed using SPSS 19.0 software (IBM, Chicago, IL, USA). The LHPP RNA expression difference between RCC tissues and matched normal tissues was analyzed by using a paired sample *t*-test. The data from the CCK-8 assay were analyzed by ANOVA. Finally, the other data were analyzed by the independent samples *t*-test. *P* value < 0.05 was considered statistically significant.

## 3. Results

LHPP was downregulated in RCC tissues, and expression was significantly associated with poor prognosis.

**Figure 1:**
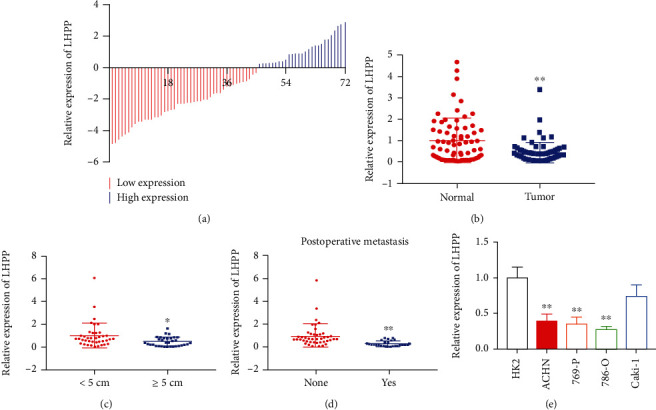
LHPP is downregulated in RCC tissues and cell lines. (a) 72 RCC samples were included in this study. The red column represents a low expression of LHPP, and the blue column represents a relatively high expression of LHPP. (b) Relative expression of LHPP in RCC tissues and matched normal tissues was shown. (c) LHPP expression in RCC tissues with different tumor sizes. (d) LHPP expression in RCC patients with or without postoperative metastasis. (e) LHPP expression was downregulated in RCC cells compared to that in HK2 cells. ^∗^*P* < 0.05 and ^∗∗^*P* < 0.01.

LHPP was augmented in RCC tissues and cell lines. The qRT-PCR assay was performed to measure CRNDE expression in RCC tissues and cell lines compared to matched normal tissues and cells. LHPP was significantly downregulated in 62.5% (45 0f 72) RCC tissues compared to adjacent normal tissues (Figure 1(a)). Total LHPP expression in matched normal tissues was 0.43 times of that in RCC tissues (Figure 1(b)). Low expression of LHPP was closely associated with tumor size and postoperative metastasis of RCC patients (Figures 1(c) and 1(d) and Table 1). Moreover, LHPP was significantly downregulated in ACHN, 769-P, and 786-O cells. Hence, we selected 786-O and 769-P as the objective of this study.

### 3.1. LHPP Inhibited the Proliferation of RCC Cells

**Figure 2:**
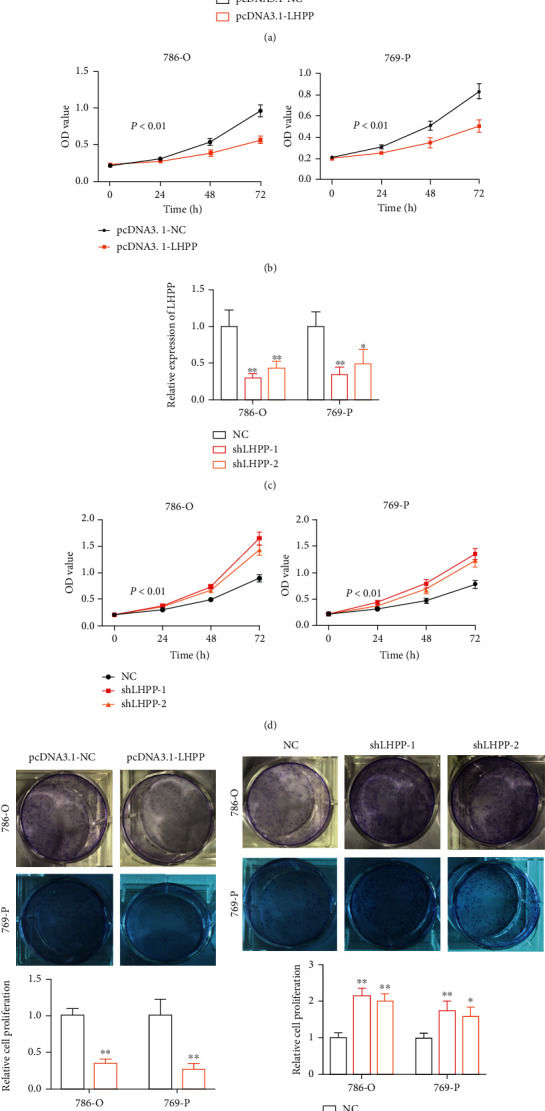
LHPP inhibits the proliferation of RCC cells. (a) The qRT-PCR assay was conducted to detect the expression of LHPP in RCC cells transfected with pcDNA3.1-LHPP. (b) Overexpression of LHPP slowed down the growth cure of RCC cells. (c) The qRT-PCR assay was conducted to detect the expression of LHPP in RCC cells transfected with shRNA-LHPP. (d) The silence of LHPP accelerated the growth cure of RCC cells. (e) Increased expression of LHPP inhibited the proliferation of RCC cells. (f) Suppression of LHPP inhibited the proliferation of RCC cells. ^∗^*P* < 0.05 and ^∗∗^*P* < 0.01.

To perform the gain or loss of function, we used pcDNA3.1-LHPP to increase LHPP expression and the shRNA-LHPP to inhibit LHPP expression. As shown in Figure 2(a), LHPP expression was significantly augmented in RCC cells when cells were transfected with pcDNA3.1-LHPP. Overexpression of LHPP slowed down the growth curve of RCC cells (Figure 2(b)). However, LHPP expression was inhibited when cells were transfected with shLHPP (Figure 2(c)). Suppression of LHPP accelerated the proliferation of RCC cells (Figure 2(d)). Furthermore, forced expression of LHPP significantly suppressed the proliferation of RCC cells (Figure 2(e)), while suppression of LHPP caused opposite effects (Figure 2(f)).

### 3.2. LHPP Inhibited the Migration of RCC Cells

**Figure 3:**
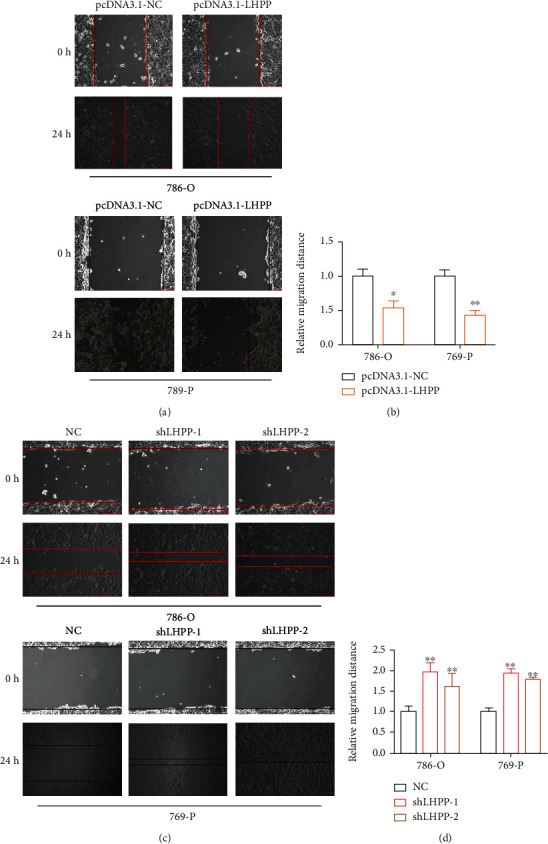
The effect of LHPP on RCC cell migration. (a, b) Overexpression of LHPP suppressed the migration of 786-O and 769-P cells. (c, d) Suppression of LHPP promoted the migration of 786-O and 769-P cells. ^∗^*P* < 0.05; ^∗∗^*P* < 0.01.

**Figure 4:**
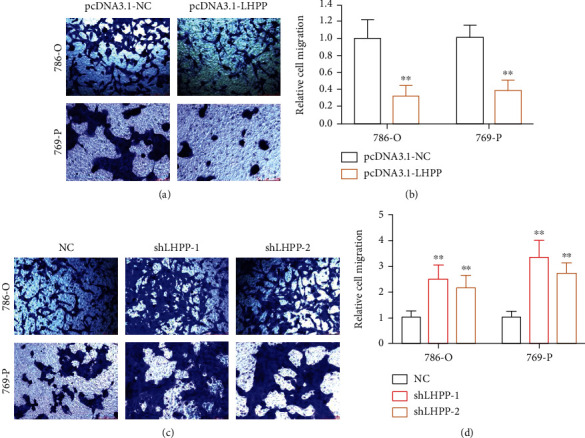
Transwell migration assay was performed to investigate cell migration. (a, b) RCC cell migration was inhibited after increasing the expression of LHPP. (c, d) RCC cell migration was enhanced after suppressing the expression of LHPP. ^∗^*P* < 0.05 and ^∗∗^*P* < 0.01.

The wound-healing assay and transwell migration assay were carried out to investigate the migration of RCC cells. The migration distance was calculated as the previous study. In the pcDNA3.1-LHPP group, the relative migration rate was decreased by 46% in 786-O and 58% in 769-P (Figures 3(a) and 3(b)). In the shLHPP group, the relative migration rate was decreased significantly in RCC cells compared with the negative control group (Figures 3(c) and 3(d)). Besides, the transwell migration assay demonstrated that forced expression of LHPP inhibited the migration of 786-O and 769-P cells (Figures 4(a) and 4(b)). However, knockdown of LHPP caused opposite effects (Figures 4(c) and 4(d)).

### 3.3. LHPP Inhibited the Invasion of RCC Cells

**Figure 5:**
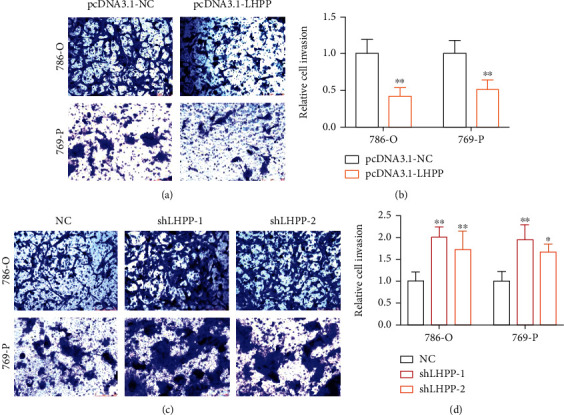
The effect of LHPP on RCC cell invasion. (a, b) RCC cell invasion was inhibited after increasing the expression of LHPP. (c, d) RCC cell invasion was enhanced after suppressing the expression of LHPP. ^∗^*P* < 0.05 and ^∗∗^*P* < 0.01.

The transwell invasion assay was performed to detect the invasion of RCC cells. The invasion abilities of RCC cells were significantly decreased by the pcDNA3.1-LHPP group. In the pcDNA3.1-LHPP group, the relative invasion rate was decreased by 59% in 786-O and 50% in 769-P (Figures 5(a) and 5(b)). In the shLHPP group, the relative migration rate was decreased significantly in RCC cells compared with the negative control group (Figures 5(c) and 5(d)).

### 3.4. LHPP Inhibited the Growth of RCC Cells

**Figure 6:**
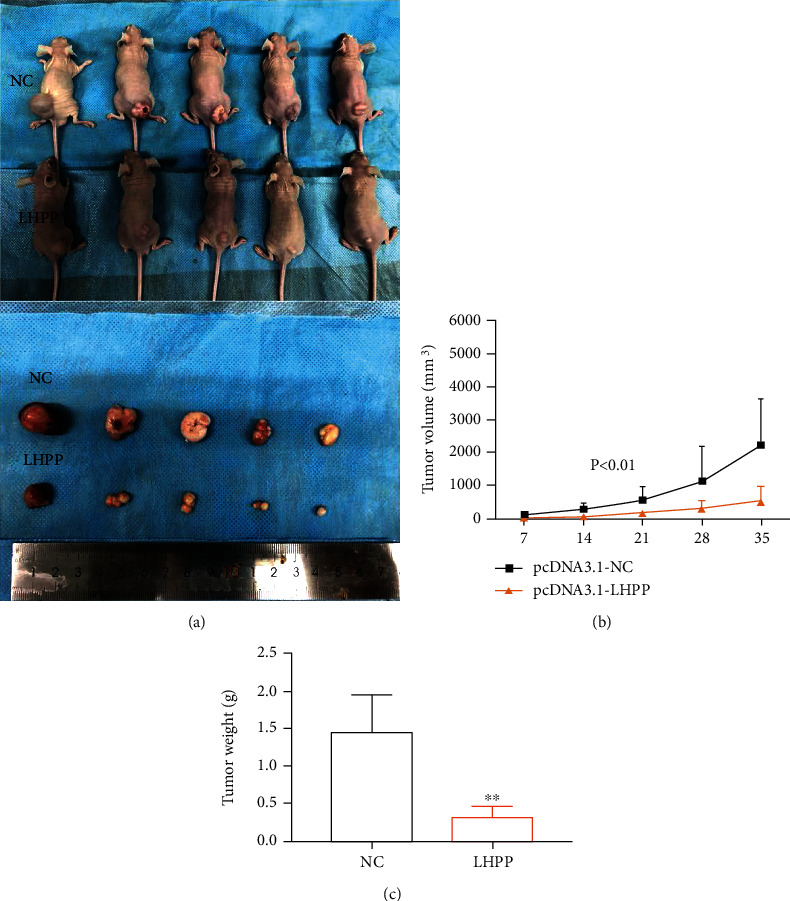
Overexpression of LHPP inhibited tumorigenesis of RCC cells in vivo. (a) Tumors derived from the LHPP group were smaller than those derived from the negative control group. (b, c) The tumors in the LHPP group were significantly decreased in volume and weight compared to the negative control group. ^∗^*P* < 0.05 and ^∗∗^*P* < 0.01.

To detect the role of LHPP in the growth of RCC cells in vivo, 786-O cells stably transfected with pcDNA3.1-LHPP or pcDNA3.1-NC were transplanted into nude mice to establish a xenograft tumor. In the xenograft tumor model, the transplanted tumors derived from 786-O cells transfected with pcDNA3.1-LHPP were much smaller than those tumors derived from cells transfected with pcDNA3.1-NC (Figure 6(a)). In addition, forced expression of LHPP significantly inhibited tumor volume as well as weight (Figures 6(b) and 6(c)).

## 4. Discussion

Renal cell carcinoma (RCC) is one of the most common tumors in the urinary system with a rising incidence rate [12]. Though aggressive treatments have improved the prognosis of RCC patients obviously, the survival rate of advanced RCC remains unsatisfied [13]. However, the molecular mechanisms involved in the tumorigenesis and metastasis that we know are only the tip of the iceberg. Therefore, it is imperative to investigate novel diagnostic markers and effective therapeutic target in RCC.

Accumulating evidence suggests that LHPP acts as a tumor suppressor during the progression of various cancers, suppressing the proliferation and metastasis of cancer cells. In bladder cancer, LHPP inhibits cell growth in vitro and in vivo via regulating the AKT/p65 signaling pathway [14]. In pancreatic cancer, LHPP suppresses cell proliferation and metastasis and promotes cell apoptosis via interacting with the PTEN/AKT signaling pathway [15]. However, the biological function of LHPP in RCC remains unknown.

In this study, we found that LHPP was significantly downregulated in RCC tissues and cell lines. Reduced expression of LHPP was positively correlated with tumor size and postoperative metastasis of RCC patients. Further experiments demonstrated that augmented expression of LHPP significantly inhibited the proliferation, migration, and invasion of RCC cells. However, suppression of LHPP causes opposite effects. In addition, overexpression of LHPP significantly inhibited the growth of RCC cells in vivo. Therefore, our results suggested that LHPP could act as a potential therapeutic target for RCC.

## Figures and Tables

**Table 1:** Correlation between LHPP expression level and clinicopathological features of renal cancer patients.

Parameters	Group	Total	LHPP expression		*P* value
			Low	High	
Gender	Male	44	30	14	0.212
	Female	28	15	13	
Age	<60	32	23	9	0.142
	≥60	40	22	18	
Clinical stage	Stage I+II	41	25	16	0.759
	Stage III+IV	31	20	11	
Tumor size	<5 cm	39	18	21	0.002
	≥5 cm	33	27	6	
Tumor stage	T1+T2	48	28	20	0.302
	T3+T4	24	17	7	
Lymph node metastasis	N0	46	31	15	0.266
	N1	26	14	12	
Postoperative metastasis	No	45	24	21	0.001
	Yes	27	21	6	

^∗^ *P* < 0.05 was considered significant (chi-square test between 2 groups).

## Data Availability

The datasets used and/or analyzed during the current study are available from the corresponding author on reasonable request.
